# Air stability of monolayer WSi_2_N_4_ in dark and bright conditions

**DOI:** 10.1038/s41598-024-73614-2

**Published:** 2024-10-06

**Authors:** Mustapha Driouech, Caterina Cocchi, Muhammad Sufyan Ramzan

**Affiliations:** 1 Institut für Physik, Carl von Ossietzky Universität, 26129 Oldenburg, Germany; 2 Center for Nanoscale Dynamics (CeNaD), Carl von Ossietzky Universität, 26129 Oldenburg, Germany

**Keywords:** Two-dimensional materials, Density functional theory, WSi_2_N_4_ monolayer, Air stability, Gas adsorption, Materials science, Nanoscience and technology, Physics

## Abstract

Two-dimensional materials with chemical formula MA_2_Z_4_ are a promising class of materials for optoelectronic applications. To exploit their potential, their stability with respect to air pollution has to be analyzed under different conditions. In a first-principle study based on density functional theory, we investigate the adsorption of three common environmental gas molecules (O_2_, H_2_O, and CO_2_) on monolayer WSi_2_N_4_, an established representative of the MA_2_Z_4_ family. The computed adsorption energies, charge transfer, and projected density of states of the polluted monolayer indicate a relatively weak interaction between substrate and molecules resulting in an ultrashort recovery time of the order of nanoseconds. O_2_ and water introduce localized states in the upper valence region but do not alter the semiconducting nature of WSi_2_N_4_ nor its band-gap size apart from a minor variation of a few tens of meV. Exploring the same scenario in the presence of photogenerated electrons and holes, we do not notice any substantial difference except for O_2_ chemisorption when negative charge carriers are in the system. In this case, monolayer WSi_2_N_4_ exhibits signs of irreversible oxidation, testified by an adsorption energy of -5.5 eV leading to an infinitely long recovery time, a rearrangement of the outermost atomic layer bonding with the pollutant, and n-doping of the system. Our results indicate stability of WSi_2_N_4_ against H_2_O and CO_2_ in both dark and bright conditions, suggesting the potential of this material in nanodevice applications.

## Introduction

The application of low-dimensional materials in real-life devices strongly depends on their interaction with environmental gas molecules under different operating conditions. The extended sp^2^ carbon conjugated network characterizing the electronic structure of graphene^1^ makes this material extremely sensitive to gas adsorption. On the one hand, this property has been exploited for sensing applications^2^. On the other hand, the interaction with atmospheric pollutants leads to p-doping^3,4^ and deteriorates the excellent carrier mobility of pristine graphene^5^. Oxidation is one of the main mechanisms appearing upon exposure of 2D materials to atmospheric environment which can significantly alter their chemical and physical properties^6–9^ and thus impact on their stability^10^. The presence of defects in the samples can additionally affect the interaction with gas adsorbates, for example, offering favorable anchoring sites^11^ and promoting chemical reactions^12,13^Similar processes occur also in the presence of water, which favorably dissociates and thus enhances sample degradation^14–17^.

The intensive research on transition metal dichalcogenide (TMDC) monolayers over the last decade^18,19^ has led to the development of related classes of novel two-dimensional materials. Among them, those with chemical formula MA_2_Z_4_, where M is a transition metal atom like Mo and W, and A and Z are nonmetallic species of group 14 and 15, respectively, has driven considerable attention in the last few years^20–23^ thanks to the synthesis and experimental characterization of two of its members, MoSi_2_N_4_ and WSi_2_N_4_^24^. Both materials are semiconductors dominated by exitonic properties^22,25^that are sensitive to the specific chemical composition^23^. These features make MoSi_2_N_4_ and WSi_2_N_4_excellent candidates for optoelectronic applications^23,26^. However, to fully disclose their potential, it is necessary to assess the air stability of these materials. While an earlier first-principle study provided corresponding insight on MoSi_2_N_4_^27^, an extension to WSi_2_N_4_is necessary to achieve a comprehensive picture, especially considering the potential alterations in adsorption behavior under light environments. This is relevant for other computationally predicted members of this family^28,29^.

First-principle methods such as density-functional theory (DFT) have significantly contributed to the development of two-dimensional materials by complementing experiments^30–33^. In particular, their ability to simulate various compositions and configurations without the aid of empirical parameters has significantly extended the range of these predictions^34–36^, stimulating new attempts for synthesis and experimental characterization^37^. Furthermore, ab initio studies contributed to shed light on the response of materials to external perturbations, including electromagnetic radiation, strain, and air pollutants with a selectivity on the number of layers and specific configuration that is hardly achievable in the lab^38–42^. The gained insight is essential to obtain an all-around understanding of the material properties, even though simulations often depict idealized scenarios compared to actual conditions.

In this work, we use DFT to investigate the air stability of monolayer WSi_2_N_4_ to three abundant atmospheric gas molecules such as O_2_, H_2_O, and CO_2_ in their vapor phase. Starting from initial configurations with the molecules sitting on different adsorption sites and assuming selected orientations with respect to the substrate, we rank the stability of the polluted systems and analyze the electronic structure of the most favorable configurations. We quantify the amount of charge transfer from the monolayer to the adsorbate and evaluate the impact of the latter on the density of states of WSi_2_N_4_. Interactions with pollutants do not introduce any significant variations in the electronic properties of the substrate, suggesting its air stability. We explored the same scenario in the presence of photogenerated electrons and holes simulated with a constrained DFT approach. The results obtained in a dark environment are substantially confirmed also under bright conditions except for the strong tendency of WSi_2_N_4_ to oxidize when an extra electron is in the environment. This phenomenon is irreversible and causes a permanent modification in the structural and electronic characteristics of the monolayer. On the other hand, this material is robust to adsorption of H_2_O and CO_2_ in both dark and bright conditions making it a good candidate for nano-devices.

## Computational methods

The results presented in this study are obtained within the framework of DFT^43^as implemented in the Vienna ab-initio simulation package (VASP)^44^employing the projector augmented wave method^45^. A 3 × 3 supercell of WSi_2_N_4_is adopted to simulate the material interacting with adsorbed gas molecules. It comprises a total of 63 atoms and a vacuum layer of about 20 Å in the non-periodic direction isolating periodic images. Structural optimization and electronic-structure calculations are performed using the generalized gradient approximation for the exchange-correlational potential as proposed by Perdew, Burke, and Ernzerhof (PBE)^46^and improved by Grimme’s D3 method^47^to account for pairwise van der Waals (vdW) interactions between gas molecules and slab. While the choice of the vdW functional is known to play an important role in the absorption of molecules on metallic substrates^48,49^, due to the larger magnitude of dispersion interactions in these systems, on semiconductors like WSi_2_N_4_variations in absorption energies and geometries obtained with different treatments of vdW interactions are negligible^50,51^. Yet, the inclusion of vdW contributions in the exchange-correlation functional is essential to obtain reliable results. Spin-orbit coupling (SOC) is included in all calculations except for the structural optimization runs. A Γ-centered 4 × 4 × 1 k-point mesh is adopted for sampling the Brillouin zone together with an energy cutoff of 500 eV and a total-energy threshold of 1 × 10^−5^ eV in the self-consistent field calculations. We carefully checked that these parameters ensure converged results. The optimized structures are obtained by minimizing interatomic atomic forces until they are below 20 meV/Å.

The charge distribution upon adsorption is evaluated using Bader charge analysis^52^ and visualized in real space through the charge density difference (CDD) defined as:

1 $$\begin{array}{c}CDD={{\uprho}}_{\text{substrate+gas}}-{({\uprho}}_{\text{substrate}}+{{\uprho}}_{\text{gas}})\end{array}$$

where ${{\uprho}}_{\text{substrate+gas}}$ is the charge density of the composite system, while ${{\uprho}}_{\text{substrate}}$ and ${{\uprho}}_{\text{gas}}$correspond to the charge density of the pristine substrate and of the isolated gas molecule, respectively. All crystal structures and CDD isosurfaces are visualized using VESTA^53^.

To mimic the presence of photogenerated electrons and holes – the so-called light environment – an electron is added or removed from the simulation cell, respectively. Under this constraint, the structures are reoptimized with the same computational parameters reported above to take into account possible atomic rearrangements.

## Results and discussion

### Structural and electronic properties of pristine WSi_2_N_4_ monolayer

**Fig. 1:**
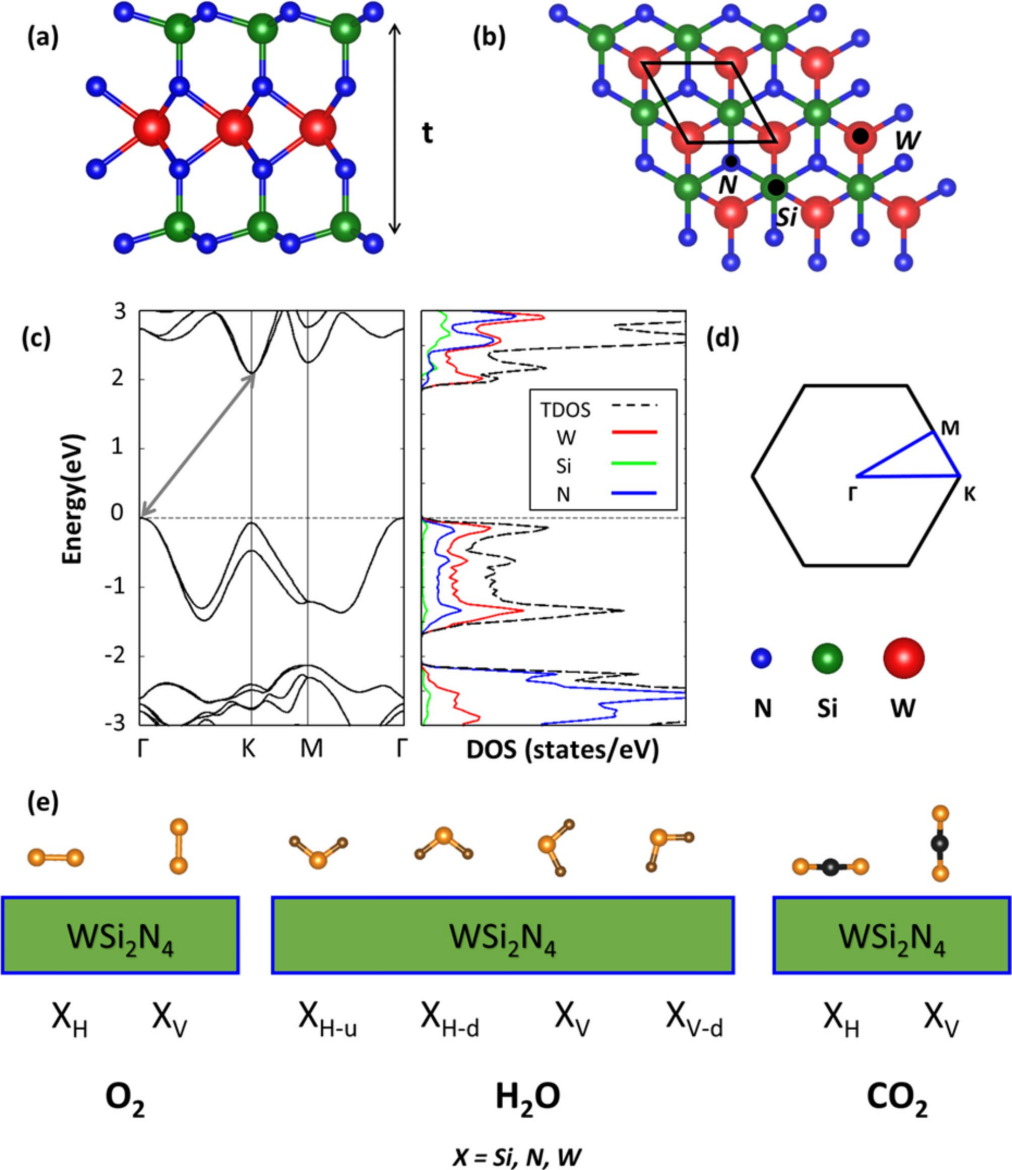
(a) Side and (b) top view of a 3 × 3 supercell of monolayer WSi_2_N_4_ with its thickness *t* marked by an arrow in panel (a) and the in-plane unit cell boundaries delimited by a black rhombus in panel (b). *N*, *Si*, and *W* indicate available adsorption sites for the gas molecules atop the corresponding atoms. (c) Electronic band structure with the fundamental gap indicated by a gray arrow (left) and projected density of states (right) of monolayer WSi_2_N_4_ described in its unit cell, with the total density of states, TDOS, shown by the dashed curve. The Fermi energy is set to zero at the VBM and marked by a horizontal dotted line. (d) Brillouin zone of WSi_2_N_4_ with the path along the high-symmetry points used to plot the band structure highlighted in blue. (e) Initial configurations of the O_2_, H_2_O, and CO_2_ molecules adsorbed on WSi_2_N_4_ on sites X *= N*, *Si*, *W* with vertical (V) or horizontal (H) arrangement with respect to the substrate. For H_2_O, two horizontal configurations with the H atoms pointing upward (H-u) or downward (H-d) are reported along with two vertical positions in which one of the O-H bonds is parallel to the substrate plane (V-d).

In the first step of our analysis, we review the structural and electronic properties of WSi_2_N_4_ in order to obtain a robust point of comparison for the subsequent investigation of the polluted systems. Monolayer WSi_2_N_4_ has a hexagonal crystal structure (space group $P\bar{6}m_{2}$)^20,23^ comprising seven atomic planes with WN_2_ groups sandwiched between two Si-N layers (Fig. 1a). Viewed from the top, this material is characterized by a hexagonal network of Si and N atoms on the uppermost layer with W atoms located on lower layers at hollow sites. This structure offers several adsorption sites for gas molecules, marked by black dots in Fig. 1b. The optimized in-plane lattice parameter (a = 2.91 Å), thickness (t = 7Å), and bond lengths (W-Si = 2.10 Å and Si-*N*= 1.75 Å) are in agreement with the literature^22,23,25,54^.

As shown in Fig. 1c, the WSi_2_N_4_ monolayer is characterized by an indirect band gap with the valence band maximum (VBM) and the conduction band minimum (CBM) at Γ and K, respectively (Fig. 1d)^22,25^. The computed band gap of 2.0 eV is in line with an earlier prediction obtained at the same level of theory^24^. It should be noticed that the adopted PBE functional typically underestimates experimental band gaps value. For example, the PBE gap of MoSi_2_N_4_ (1.74 eV) reported in Ref.^24^ is 200 meV lower than the experimental one (1.94 eV) obtained in the same study. A similar discrepancy can be expected for WSi_2_N_4_too. While more sophisticated approaches like the range-separated hybrid functional HSE^55^or the GW approximation^56^ can significantly improve the accuracy of the calculated band structure, they demand very high computational costs especially for complex systems such as those considered hereafter. Since this work is primarily focused on assessing the air stability of WSi_2_N_4 _and several works have confirmed the ability of PBE to provide a qualitatively reliable description of the electronic structure of analogous systems^22,41^in the context of gas adsorption^27,57–61^, we can confidently continue our analysis using this functional.

The band structure of monolayer WSi_2_N_4_ reported in Fig. 1c reveals that, in the valence region, the valley at K point is only 76 meV below the VBM at Γ and split off by 403 meV. SOC due to W atoms is responsible for this behavior which appears also in the band structure of WS_2_ and WSe_2_^54,62,63^. The analysis of the projected density of states (PDOS, Fig. 1c) indicates that both the VBM and the CBM have predominant contributions from the W atoms. In the valence, hybridization with N is present in the two uppermost bands split by SOC around K with almost negligible contributions from Si. At lower energies, N states prevail with participation from W and, to a smaller extent, Si. In the conduction region, contributions from W and N atoms are almost equal above the CBM with the Si orbitals gaining weight at higher energies.

### Gas adsorption on WSi_2_N_4_ monolayer in a dark environment

**Fig. 2:**
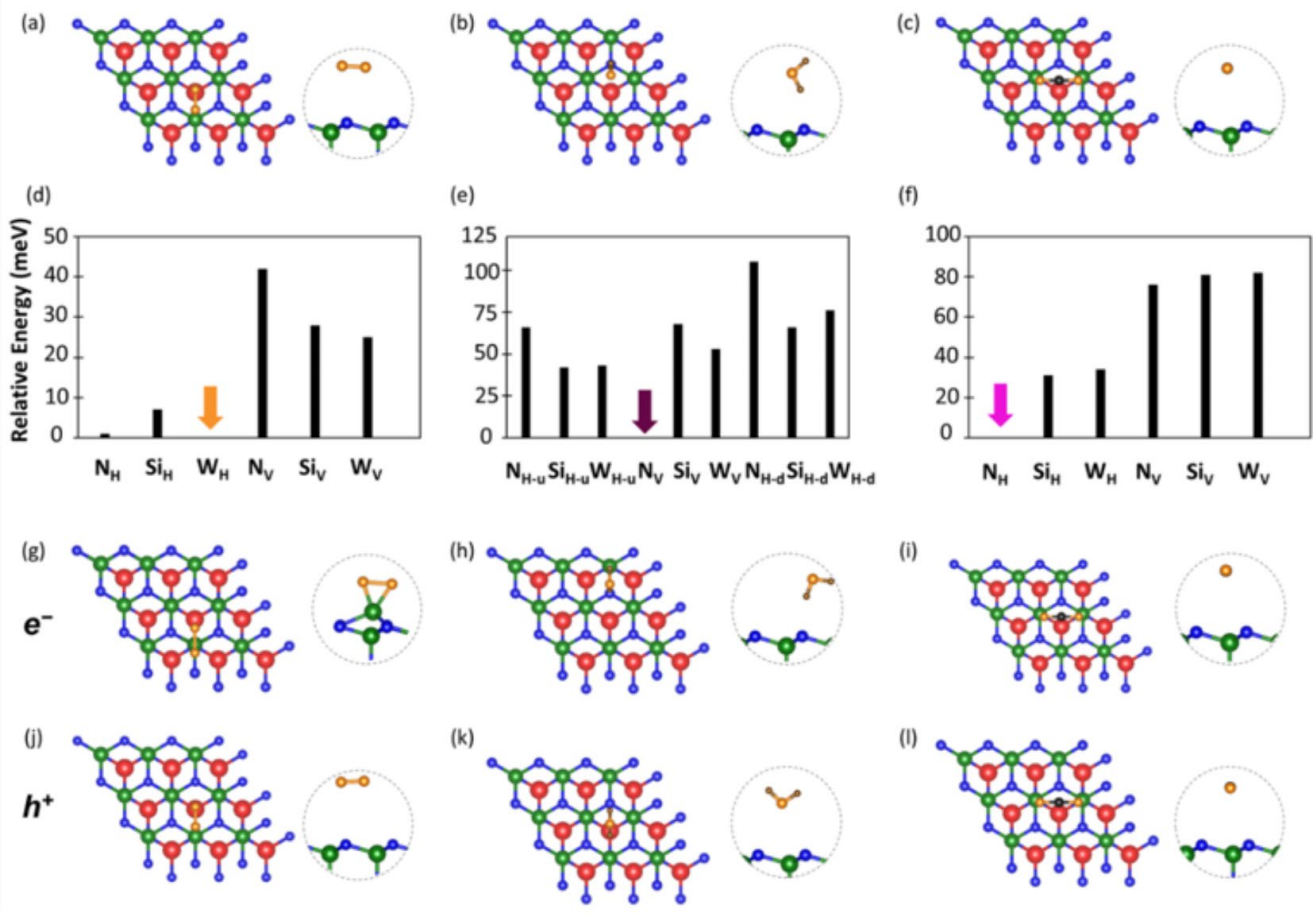
Optimized crystal structures of (a) O_2_, (b) H_2_O, and (c) CO_2_ adsorbed on monolayer WSi_2_N_4_ in the dark environment. Relative energies of (d) O_2_, (e) H_2_O, and (f) CO_2_ adsorbed on WSi_2_N_4_ in all considered configurations computed with respect to the most stable one set to zero and marked by a colored arrow. Optimized structures in the presence of photogenerated electrons for (g) O_2_, (h) H_2_O, and (i) CO_2_ adsorbed on monolayer WSi_2_N_4_. Optimized crystal structures in the presence of photogenerated holes for (j) O_2_, (k) H_2_O, and (l) CO_2_ adsorbed on monolayer WSi_2_N_4_. The insets show a magnified view of the adsorbed molecule and of the uppermost atomic layer of the substrate.

In the next step of our analysis, we investigate the adsorption of three common gas molecules, O_2_, CO_2_, and H_2_O, on monolayer WSi_2_N_4_. It is worth noting that despite its large abundance in atmosphere, we do not include N_2_ in our analysis: due to its inert character, this molecule is not expected to have a significant impact on the intrinsic properties of the 2D substrate. Each considered moiety is initially positioned at the sites highlighted in Fig. 1b with various orientations with respect to the substrate (see Fig. 1e). For the linear molecules O_2_ and CO_2_, starting configurations correspond to the adsorbates aligned horizontally (H) or perpendicularly (V) to the monolayer. In the case of H_2_O, four orientations are explored, including the horizontal alignment of the molecule with respect to the substrate with H atoms pointing upwards (H-u) or downwards (H-d), as well as the vertical alignment (V) including a variant with one O-H bond parallel to the surface (V-d). After optimizing all the considered adsorbed configurations, we rank their stability in terms of relative energy computed with respect to the most stable structure. Upon O_2_ adsorption, the most favorable configuration corresponds to the molecule aligned horizontally on the *W* site (Fig. 2a), while both H_2_O and CO_2_ are adsorbed vertically on top of N atoms (Fig. 2b-c). We emphasize that results reported herein include only energetic contributions at zero temperature. For this reason, we cannot guarantee that the identified minima correspond to global minima on the Boltzmann distribution. In order to assess this point quantitatively, it is necessary to perform molecular dynamics simulations, which, however, go beyond the scope of this work.

The relative stability of all the considered configurations is plotted in Fig. 2d-f, where the energies of the most favorable structures is set to zero for reference and indicated by colored arrows. In examining these results, we notice the different energy scale associated with the three graphs. In Fig. 2d, the energy difference between the most stable configuration and the other ones spans a range of less than 50 meV. The energetic variations among the horizontal arrangement of the O_2_ molecule adsorbed on the three considered sites are below 10 meV with the difference between *W*_*H*_ and *N*_*H*_ (*Si*_*H*_) being only 1 meV (7 meV). The vertical adsorption of O_2_ is less favorable by one order of magnitude, with energy differences with respect to the most stable configuration being 25 meV (*W*_*V*_), 28 meV (*Si*_*V*_), and 42 meV (*N*_*H*_). These results are consistent with the adsorption energies obtained at the same level of theory for the same molecules on MoSi_2_N_4_^27^.

Upon H_2_O adsorption, the situation is more faceted. Not only does this molecule offer more orientational degrees of freedom compared to the other two, but also the energetic landscape of the resulting configurations is more varied. The most stable structure with H_2_O adsorbed vertically on top of N (Fig. 2b) is clearly favored over all the others: the energy difference with respect to the system hosting H_2_O at the *Si*_*H−u*_ site is as high as 42 meV (Fig. 2e). For comparison, the least favored arrangement with H_2_O adsorbed on top of a N atom (*H-d* site) leads to an adsorption energy that is > 100 meV larger than the most stable structure.

For CO_2_, we find a clear trend, similar to the one obtained for the other linear molecule O_2_. As illustrated in Fig. 2f, the most stable structure emerges upon adsorption on top of N with the axis of the molecule aligned horizontally with respect to the substrate (see Fig. 2c, where only one O atom is visible as the axis of the molecule is perpendicular to the visual plane). The adsorption of CO_2_ aligned horizontally with respect to WSi_2_N_4_ is clearly favored for all considered adsorption sites. The energy difference among the three configurations is below 40 meV, with almost identical energies obtained at *Si*_*H*_ and *W*_*H*_ sites (Fig. 2f). Similarly, the adsorption of CO_2_ in the vertical arrangement is unfavored by about 80 meV compared to the most stable structure and is rather insensitive of the adsorption site.

We evaluate the adsorption energy (E_ads_) of the gas molecules in the most favorable configurations as

2 $$\begin{array}{c}{E}_{\text{ads}}={E}_{\text{substrate+gas}}-{E}_{\text{substrate}}-{E}_{\text{gas}},\end{array}$$

where ${E}_{\text{substrate+gas}}$ represents the total energy of the substrate with the adsorbed gas molecule, while ${E}_{\text{substrate}}$ and ${E}_{\text{gas}}$ are the energies of the isolated substrate and gas molecule, respectively, computed in the same supercell as the composite system. The adsorption energies for the most preferred configurations are on the order of -0.20 eV, see Table 1. The distance between the gas molecules and the substrate varies from system to system although some general trends can be identified. Since both O_2_ and CO_2_ preferentially adsorb horizontally with respect to the underlying substrate, they exhibit a similar separation of about 3 Å from the WSi_2_N_4_ monolayer. The smaller distance obtained for O_2_ (2.97 Å) compared to the one for CO_2_ (3.09 Å) is most likely due to the adsorption site: the W atom lying on a deeper atomic layer exerts less repulsion than interfacial N. On the other hand, the adsorbed H_2_O molecule with H atoms pointing to the WSi_2_N_4_ substrate, remains at a distance of 2.14 Å from it. This value can be understood in terms of electrostatic attraction between the H atoms and the N atom on top of which the molecule is adsorbed.

**Table 1:** Adsorption site and corresponding adsorption energy (E_ads_), molecule-substrate distance, charge transfer (Δq), shift of the band gap with respect to the isolated WSi_2_N4 monolayer ($\varDelta$**E**_**gap**_), and recovery time $\tau$ at room temperature (300 K) and at an elevated temperature (500 K) computed for the most stable configuration for adsorbed O_2_, H_2_O, and CO_2_ molecules.

Gas	Ads. site	E_ads_ (eV)	d_sub-gas_ (Å)	Δq (e)	$\varDelta$E_gap_ (eV)	τ_300K_ (ns)	τ_500K_ (ns)
O_2_	*W*_*H*_	-0.19	2.97	0.12	0.13	1.5	0.08
H_2_O	*N*_*V*_	-0.19	2.14	0.03	0.06	1.5	0.08
CO_2_	*N*_*H*_	-0.20	3.09	0.02	0.05	2.0	0.09

To evaluate the influence of gas adsorption on the intrinsic properties of the WSi_2_N_4_ substrate, the recovery time $\tau$ can be computed. This quantity represents the time required by the material to be restored in its initial configuration. The recovery time depends exponentially on the adsorption energy ${E}_{\text{ads}}$ according to the formula

3 $$\begin{array}{c}\tau={v}^{-1}\cdot\text{exp}\left(\frac{{E}_{\text{ads}}}{{k}_{B}T}\right),\end{array}$$

where $v$ is the attempt frequency of bond breaking, ${k}_{B}$ the Boltzmann constant, and $T$ the temperature. A value of $v=1\,THz$ is adopted here, as reported in earlier experimental and theoretical studies^58,64,65^. The recovery times reported in Table 1 for the three most stable configurations of O_2_, H_2_O, and CO_2_ adsorbed on WSi_2_N_4_ are on the order of nanoseconds at room temperature (300 K) and even shorter (around 80 ps) at 500 K. These outcomes indicate that WSi_2_N_4_ can quickly recover its initial condition after the adsorption of the aforementioned pollutants. We can briefly compare the recovery times estimated for O_2_, H_2_O, and CO_2_ adsorbed on WSi_2_N_4_ with results obtained at the same level of theory for related 2D materials. On MoSi_2_N_4_, the recovery time estimated at room temperature for water is the same as found here (~ 1.5 ns) while it is one and two orders of magnitude lower for CO_2_ and O_2_, respectively^27,66^. Recovery times on the order of tens of picoseconds are also predicted for CO_2_ and O_2_adsorbed on Mo-based TMDCs at 300 K^67^. While no experimental measurements are yet available for WSi_2_N_4_, the comparison with experimental data is in general not straightforward, due to the crucial role played by the details and parameters of the device in assessing gas adsorption performance.

**Fig. 3:**
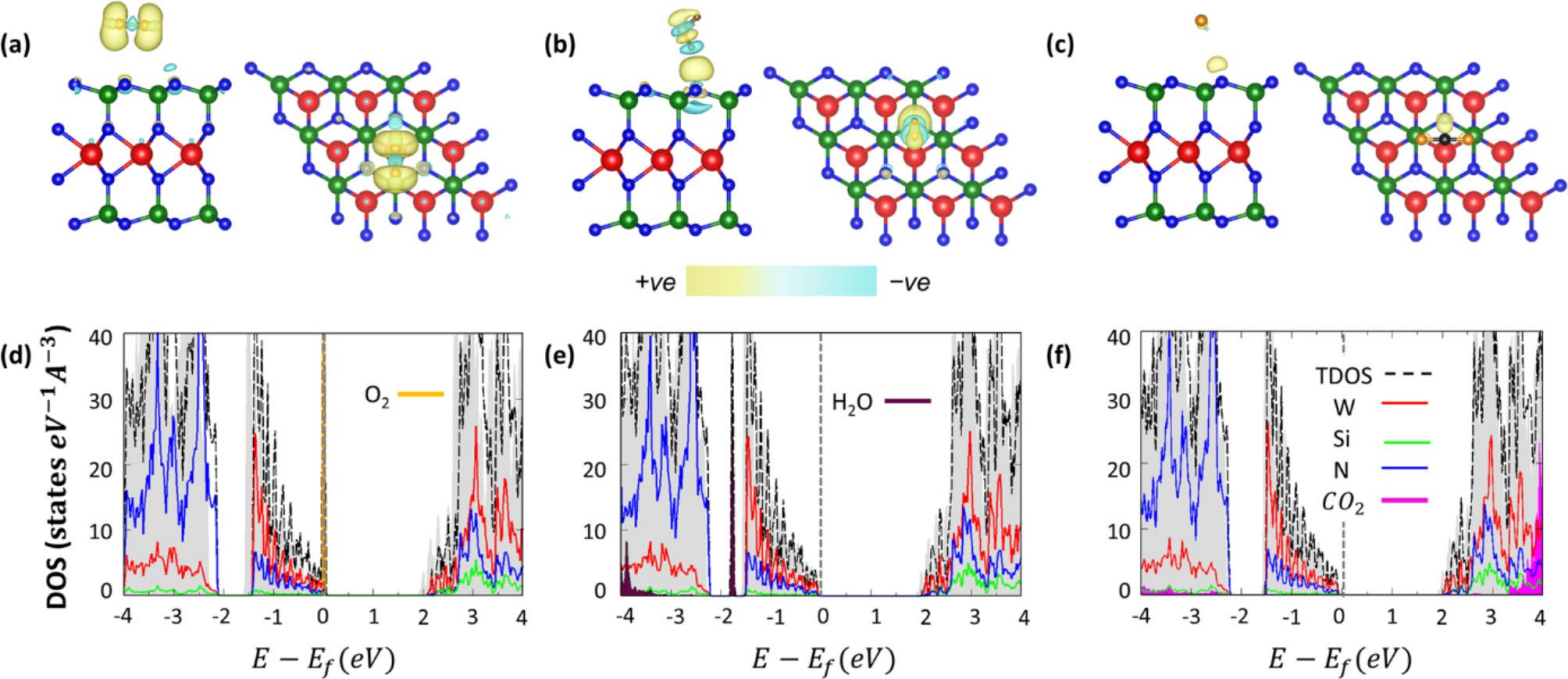
Top and side views of the CDD of the energetically most stable configuration of (a) O_2_, (b) H_2_O, and (c) CO_2_ adsorbed on monolayer WSi_2_N_4_. Electronic charge accumulation (+ ve) and charge depletion (-ve) is indicated in yellow and cyan, respectively. An isosurface value of 0.0005 eA^-3^ is adopted in all CDD plots. Total density of states (TDOS) and projected atomic and molecular contributions in the composites systems including (d) O_2_, (e) H_2_O, and (f) CO_2_ adsorbed on WSi_2_N_4_. The PDOS of the isolated substrate is displayed for comparison (gray area). The Fermi energy (E_f_) is set to zero at the VBM and marked with a vertical dashed line. For better visualization, the contributions of CO_2_ in panel (f) are magnified by a factor 20.

In the next step of our analysis, we discuss the electronic properties of the three most stable configurations with gas molecules adsorbed on monolayer WSi_2_N_4_. We start by inspecting the charge distribution obtained from Bader charge analysis (Table 1) and visualized through the charge density distribution (CDD). The positive sign of the charge difference Δq between the isolated constituents and the adsorbed systems indicates that electronic charge is transferred from the substrate to the gas molecules. O_2_ withdraws significantly more charge compared to the other two molecules: Δq = 0.12 e vs. 0.02 e by CO_2_ and 0.03 e by H_2_O. This behavior is consistent with the picture provided by the CDD in Fig. 3a, where considerable charge accumulation is localized on the two oxygen atoms. Charge depletion domains in the top-most atomic layer of WSi_2_N_4_ of this composite system confirms the electronic withdrawal of the adsorbate from the substrate especially around the interfacial N atoms. The CDD plot obtained for the H_2_O-adsorbed monolayer (Fig. 3b) exhibits alternated domains of charge accumulation and depletion. Most of them appear at the interface between molecule and surface, explaining the relatively low amount of charge localized on the former. Again, the uppermost N atoms of WSi_2_N_4_ appear particularly involved in the charge-transfer process, as expected, recalling that H_2_O is adsorbed on top of N. Finally, the adsorption of CO_2_ generates a minimal charge transfer of 0.02 e, visualized in Fig. 3c by charge accumulation domains mostly distributed in the interfacial region on top of N atoms.

With the aid of the density of states (DOS), we analyze how the adsorbed moieties influence the electronic structure of the underlying monolayer, see Fig. 3d-f, where the result obtained for the most stable configurations with O_2_, H_2_O, and CO_2_ (dashed black curves) are contrasted against the DOS of isolated WSi_2_N_4_ (gray area, same as in Fig. 1c). Gas adsorption influences the band-gap size of the substrate to a small but non-negligible extent. Specifically, the presence of O_2_ reduces the gap of WSi_2_N_4_ by 130 meV (see also Table 1), due to the upshift of the conduction band (Fig. 3d). Both manifolds of valence bands visualized in Fig. 3d, namely the top one between the VBM and − 1.5 eV and the one starting around − 2 eV, are upshifted by the same amount of energy with respect to the DOS of the isolated monolayer. Upon adsorption of H_2_O and CO_2_, the band gap of the system increases by 50 meV and 60 meV, respectively, with respect to pristine WSi_2_N_4_ (Table 1), again owing to the energetic upshift of the conduction bands, see Fig. 3e (H_2_O) and Fig. 3f (CO_2_).

Focusing now on the contributions from the PDOS, we notice that the O_2_ introduces a localized state at the top of the valence band of monolayer WSi_2_N_4_, as upon adsorption on MoSi_2_N_4_^27^, leaving the remaining features unaltered (Fig. 3d). H_2_O gives rise to a sharp peak in the occupied region of the PDOS about 2 eV below the VBM (Fig. 3e). Additional contributions to the valence region appear deeper in energy, about 4 eV below the VBM. Adsorbed CO_2_ introduces contributions both in the valence region within the band manifold starting around − 2.5 eV where a maximum around − 3.5 eV can be identified (see Fig. 3f), and especially in the conduction band, where a sharp peak appears between 3.5 and 4 eV. Note that these features are magnified by a factor of 20 in Fig. 3f for better visibility.

### Gas adsorption on monolayer WSi_2_N_4_ in a bright environment

To complete our analysis, we explore the behavior of monolayer WSi_2_N_4_ absorbing O_2_, H_2_O, and CO_2_ molecules in a bright environment simulated with the constrained DFT approach described in the “Computational methods” section. As a starting point for these calculations, we assume the most favorable adsorption site for the molecules in the dark environment, see Table 1, and let the composite systems relax under bright conditions.

**Table 2:** Bright environment and corresponding adsorption energy (E_ads_), molecule-substrate distance, charge transfer (Δq), shift of the band gap with respect to the isolated WSi_2_N4 monolayer ($\varDelta$**E**_**gap**_), and recovery time $\tau$ at room temperature (300 K) and at elevated temperature (500 K) computed for the most stable configuration with oxygen, water, and carbon dioxide molecules in the presence of photogenerated electrons and holes.

Gas	env.	E_ads_ (eV)	d_sub−gas_ (Å)	Δq (e)	$\varDelta$E_gap_ (eV)	τ_300K_ (ns)	Τ_500K_ (ns)
O_2_	e^−^	-5.51	1.48	1.299	-0.30	3.6E + 88	3.4E + 36
	h^+^	-0.17	3.33	0.008	0.03	0.8	0.05
H_2_O	e^−^	-0.18	2.13	0.025	0.10	1.0	0.07
	h^+^	-0.12	2.76	0.003	0.06	0.1	0.02
CO_2_	e^−^	-0.20	3.08	0.015	0.10	1.7	0.1
	h^+^	-0.20	3.08	0.017	0.08	2.0	0.1

**Fig. 4:**
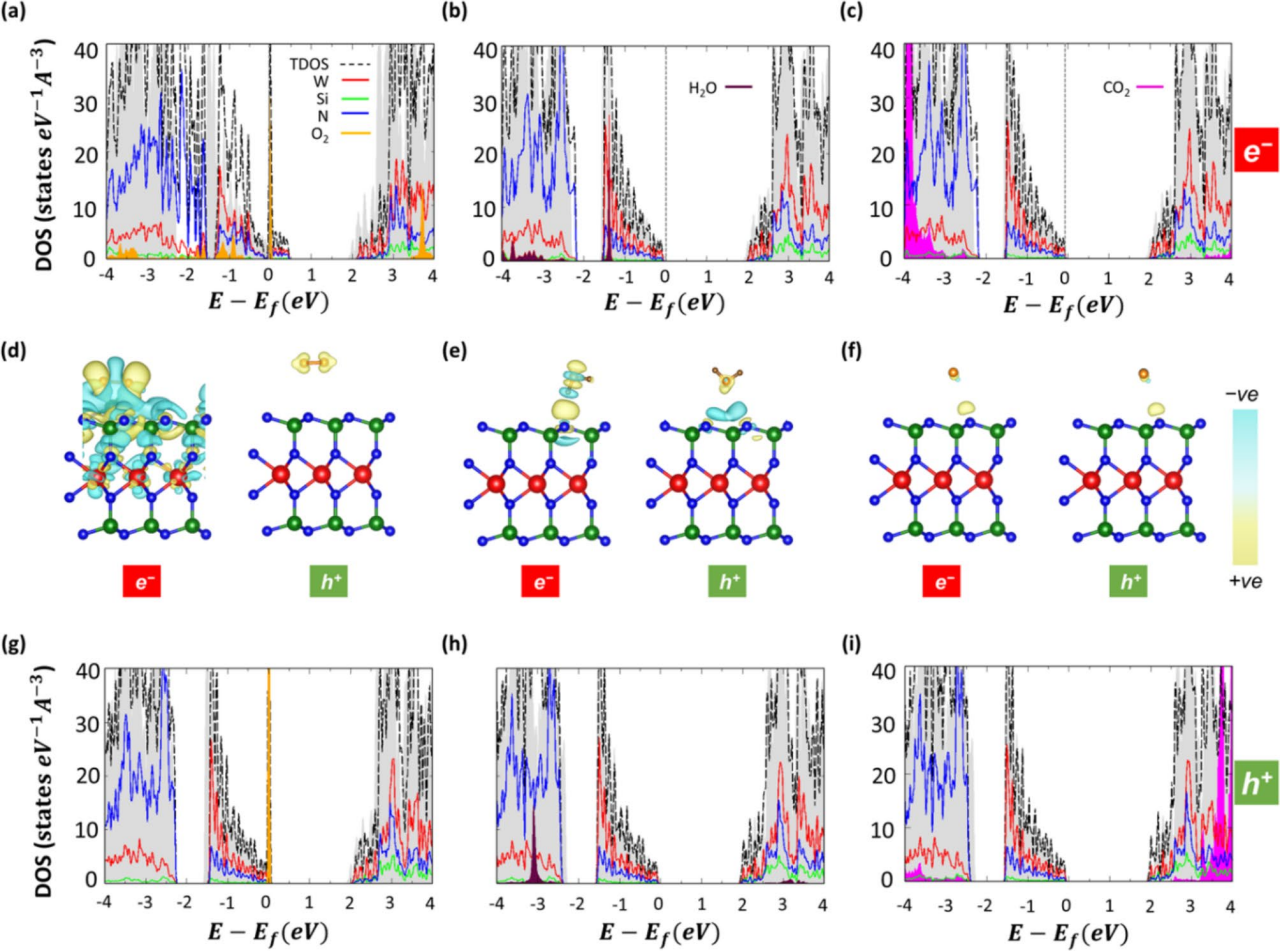
Electronic structure of O_2_, H_2_O, and CO_2_ molecules absorbed on the monolayer WSi_2_N_4_in a bright environment. (a – c) PDOS of the aforementioned systems computed in the presence of photogenerated electrons, (d – f) charge density difference with extra electrons (left) and holes (right) depicted with an isosurface value of 0.0005 eA^-3^ with charge accumulation (+ ve) in yellow and charge depletion (-ve) in cyan, and (g – i) PDOS in the presence of photogenerated holes. In the PDOS plots, the Fermi level is set to zero at the VBM, except for panel (a) where the system is n-doped, and marked by the vertical dashed line. For better visualization, the PDOS contributions of CO_2_ are magnified by a factor 20.

From the inspection of Table 2, we identify at a glance one scenario that differentiates substantially from the others and from the picture obtained in dark: in the presence of a photogenerated electron, the adsorption energy of O_2_ becomes as large as -5.51 eV, signalizing a chemical bond formation between molecule and substrate. In fact, the distance between O_2_ and the underlying WSi_2_N_4_ monolayer reduces to 1.48 Å becoming approximately half of the separation between the same molecule and the substrate in the absence of photogenerated charge carriers (see Table 1). Furthermore, the interfacial atomic layer in WSi_2_N_4_ is locally altered by O_2_ adsorption (Fig. 2g), due to the displacement of Si atom forming a bond with O_2_. As a consequence, a significant amount of charge (1.3 e) is transferred from WSi_2_N_4_ to O_2,_ and the system is n-doped (Fig. 4a). Correspondingly, the CDD plot indicates a substantial charge redistribution in the adsorbed system in the presence of a photogenerated electron, involving not only the interfacial layer but also the deeper lying W atoms (Fig. 4d). All these features point to substrate oxidation, which is further confirmed by the infinitely long recovery time obtained at room temperature (300 K) as well as at elevated temperature (500 K) suggesting that this phenomenon is irreversible.

When the environment contains photogenerated holes, the response of monolayer WSi_2_N_4_ to oxygen adsorption is analogous to dark conditions: O_2_ lies with the molecular axis aligned horizontally with respect to the substrate on the W hollow site and no bonds is formed with the substrate (Fig. 2j). The adsorption energy amounts to -0.18 eV (Table 2) and the separation between adsorbate and substrate increases by about 0.4 Å in the presence of a photogenerated hole, reaching 3.33 Å (Table 2). In this case, there is a negligible charge transfer (0.008 e) between O_2_ and WSi_2_N_4_, which is even smaller than the counterpart in dark although the CDD plots are visually identical (compare Figs. 3a and 4d). The band gap is almost unaffected by the presence of holes compared to the counterpart in dark nor significant differences appear in the PDOS, where a molecular orbital from O_2_ appears at the VBM (Fig. 4g). The recovery time at both 300 K and 500 K decreases by approximately a factor of 2 in the presence of holes compared to the results obtained in dark, which is on the order of nanoseconds and tenths of nanoseconds, respectively (compare Tables 1 and 2).

The adsorption of H_2_O on monolayer WSi_2_N_4_ in a bright environment is energetically as favorable as in dark conditions (compare Tables 1 and 2) except that the presence of holes leads to a decrease in the adsorption energy down to -0.12 eV. The preferred adsorption site remains the same, namely atop an N atom and the distance between adsorbate and substrate follows the same trend as in the dark. With an extra electron in the system, this separation remains the same as in dark conditions (2.13 Å), although the molecule undergoes an approximately 30° rotation (Fig. 2h) compared to its configuration in dark. In the presence of a hole, the substrate-molecule separation increases to 2.76 Å, and H_2_O undergoes a substantial reorientation with one oxygen atom pointing downward to the substrate. In this configuration, absorption is favored on the *W* hollow site (Fig. 2k) but the repulsion from interfacial N atoms leads to a larger separation from the substrate compared to the configuration where the H_2_O molecule is aligned vertically. Charge transfer increases by one order of magnitude in the presence of an extra electron compared to the system in dark or with an extra hole (Table 2). On the other hand, while the fundamental gap remains essentially unchanged in dark and bright environments, there are significant differences in the PDOS obtained with and without photogenerated charge carriers. In the presence of an extra electron, the localized state associated with the adsorbed H_2_O molecule remains in the valence region, as in dark conditions, but it shifts up to approximately − 1.5 eV with respect to the VBM (Fig. 4b). In contrast, when a hole is present, the occupied orbitals of H_2_O appear much deeper in energy, beyond the range visualized in Fig. 4h. Even the CDD plot is affected by the presence of photogenerated electrons and holes: in the former case, the result is visually very similar to the one obtained in dark, whereas with an extra positive charge carrier, the amount of charge density at the interface is reduced – also due to the larger molecule-substrate distance – with charge accumulation (depletion) domains on the adsorbate (monolayer), see Fig. 4e. The recovery time estimated in the presence of photogenerated holes at 300 K and 500 K remains on the same order as in dark (1.0 ns and 0.07 ns) while with an extra electron in the environment these values decrease by an order of magnitude being 0.1 ns at 300 K and 0.02 at 500 K. Overall, these results suggest that the WSi_2_N_4_ substrate returns in its pristine conditions in an ultrashort time window.

The adsorption of CO_2 _in a bright environment is very similar to the same process in dark. While this molecule is known to bend in the presence of excess electrons (see, e.g., Ref^68^), this behavior cannot be captured within the adopted theoretical approach, simulating the additional charge with a jellium model. All quantities displayed in Table 2 are (almost) identical to those reported in Table 1 and, likewise, no significant differences are noticed in the presence of photogenerated electrons or holes. The molecule lies preferentially with its horizontal axis parallel to the substrate and atop an N atom (see Fig. 2i and l). The adsorption energy is -0.2 eV in all cases and the distance between adsorbate and substrate remains of approximately 3.1 Å. The recovery times computed at both 300 K and 500 K are on the order of 0.1 ns The amount of charge transfer does not change either (0.02 e) and the CDD plots reported in Fig. 4f are visually indistinguishable from those computed in dark, see Fig. 3c. The band gap increases only by 10 meV (8 meV) with an extra electron (hole) compared to the dark conditions. On the other hand, the CO_2_ contributions to the PDOS are visibly different with photogenerated charge carriers in the environment compared to adsorption in dark conditions. In the presence of an extra electron, CO_2_ occupied orbitals upshift in energy appearing around − 4 eV in the PDOS (Fig. 4c). In the conduction region, the sharp peak characterizing the electronic structure of CO_2_-adsorbed WSi_2_N_4_ no longer appears in the PDOS shown in Fig. 4c suggesting an energy increase of this state. Conversely, this feature is downshifted compared to the PDOS for the system adsorbed in dark, appearing between 3.5 and 4.0 eV in Fig. 4i. The valence region reported in that plot is very similar to the one computed in dark. The recovery times at 300 K and 500 K obtained in the presence of an extra hole (2.0 ns and 0.1 ns, respectively) are almost identical to those obtained in dark while with a photogenerated electron in the system, there is a slight decrease of the value at room temperature (1.7 ns) while at 500 K $\tau$ remains in the order of 0.1 ns (Table 2). These findings suggest that in none of the explored scenarios CO_2_ perturbs the WSi_2_N_4_ substrate beyond an ultrashort time window, confirming the stability of this material with respect to CO_2_ pollution.

## Summary and conclusions

In summary, we reported a first-principle analysis of the air stability of monolayer WSi_2_N_4_ in dark and bright conditions. We investigated the adsorption of three abundant gas molecules (O_2_, H_2_O, and CO_2_) on different sites and with varying initial orientations. We energetically ranked the obtained configurations and focused on the most stable structures in the subsequent analysis of the adsorption energy, charge transfer, and electronic structure. In the dark environment, namely when gas adsorption occurs in the ground state, all molecules bind with an approximately equal strength of -0.20 eV. O_2_ and CO_2_ are adsorbed with their axis aligned horizontally with respect to the substrate atop W and N atoms, respectively. On the other hand, H_2_O sits preferentially in a vertical configuration on top of N. All adsorbates withdraw charge from the monolayer: lower values of 0.02–0.03 e are obtained for H_2_O and CO_2_ in comparison to 0.12 e for O_2_ due to its larger interaction with the substrate. In the electronic structure of the composite systems, all adsorbates lead to a slight increase of the band gap of WSi_2_N_4_ on the order of tens of meV. O_2_ and H_2_O introduce molecular orbitals in the valence region of the substrate while CO_2_ states do not contribute to the gap region of the latter. The recovery times computed for all these configurations at 300 K and 500 K are in the order of nanoseconds or less, suggesting that the WSi_2_N_4_ is altered by the presence of pollutants only over an ultrashort time window.

This scenario is substantially preserved in the presence of photogenerated holes. The only difference is that under these conditions, CO_2_ unoccupied states downshift in energy approaching the bottom of the conduction region of WSi_2_N_4_ and H_2_O no longer introduces a localized orbital in the uppermost valence region of the substrate. Moreover, in this case, the H_2_O molecule is most favorably adsorbed with one oxygen atom pointing downward to the hollow site atop W. In this configuration, the distance from the monolayer increases and, concurrently, charge transfer decreases. The presence of photogenerated electrons leaves the scenario almost unaltered for H_2_O and CO_2_ compared to the dark conditions, expect for an upshift of occupied levels of the latter molecule leading to sharp peak in the uppermost valence region of the PDOS of the composite system. On the other hand, significant changes occur upon O_2_ adsorption. The inclusion of extra electrons in the environment contributes to the oxidation of the substrate. The adsorption energy becomes as high as -5.5 eV, the separation between the molecule and WSi_2_N_4_ reduces to ~ 1.5 eV, and the gas pollutant withdraws 1.3 e from the monolayer. The electronic structure of the latter exhibits signatures of n-doping with the molecular orbital of O_2_ appearing in the upper portion of the valence band but not at the maximum. The recovery time reveals that this oxidation process is irreversible.

In conclusion, the results presented in this work indicate that monolayer WSi_2_N_4_ is stable upon air pollution in dark conditions. In this case, O_2_, H_2_O, and CO_2_ withdraw only a very small fraction of charge from the substrate, indicating a very similar scenario to the one predicted for MoSi_2_N_4 _at the same level of theory^27^. H_2_O and especially O_2_ influence the electronic structure of WSi_2_N_4_ by introducing localized states in the valence region and at the VBM, respectively while significant contributions from CO_2_ appear only deeper in the valence and conduction region in the presence of extra electrons and holes, respectively. Oxidation of WSi_2_N_4_ is induced by O_2 _adsorption with photogenerated electrons suggesting that under these conditions, the sample should be kept in an oxygen-poor environment to prevent an irreversible alteration of its structural and electronic properties. The introduction of a capping layer like hBN^69^, established in TMDC-based devices, represents a viable strategy. It is worth noting that chemisorption of O_2_ adsorbed on MoSi_2_N_4 _was predicted even in dark conditions when defects are present in the substrate^27^. On the other hand, neither H_2_O nor CO_2_ damage the system under bright conditions revealing a considerable stability of monolayer WSi_2_N_4_ with respect to these pollutants.

The outcomes of this study are expected to stimulate a considerable body of future research, for example, in the direction of assessing the role of thermodynamics and dissociation mechanisms in the adsorbed molecules. Likewise, for a more realistic modelling of atmospheric conditions, the simultaneous absorption of more than one gas species should be taken into account. Also, understanding the air stability of the wet monolayer is another important point to address in upcoming studies. The role of defects and edges in the 2D materials and their nanostructures is another important aspect to be explored. Finally, the simulation of bright conditions in the framework of real-time time-dependent DFT in conjunction with Ehrenfest dynamics is another interesting direction to explore in the near future.

## Data Availability

The data that support the findings of this study are available on the Zenodo database with DOI: 10.5281/zenodo.13269390.

## References

[1] Castro Neto, A. H., Guinea, F., Peres, N. M. R., Novoselov, K. S. & Geim, A. K. The electronic properties of graphene. *Rev. Mod. Phys.* **81**, 109–162 (2009).

[2] Rumyantsev, S., Liu, G., Shur, M. S., Potyrailo, R. A. & Balandin, A. A. Selective gas sensing with a single pristine graphene transistor. *Nano Lett.* **12**, 2294–2298 (2012). 22506589 10.1021/nl3001293

[3] Xu, W. *et al.* N-doped graphene field-effect transistors with enhanced electron mobility and air-stability. *Small* **10**, 1999–2005 (2014). 24616289 10.1002/smll.201303768

[4] Peng, Z., Yang, R., Kim, M. A., Li, L. & Liu, H. Influence of O2, H2O and airborne hydrocarbons on the properties of selected 2D materials. *RSC Adv.* **7**, 27048–27057 (2017).

[5] Yang, Y., Brenner, K. & Murali, R. The influence of atmosphere on electrical transport in graphene. *Carbon* **50**, 1727–1733 (2012).

[6] Habib, T. *et al.* Oxidation stability of Ti3C2Tx MXene nanosheets in solvents and composite films. *npj 2D Mater. Appl.* **3**, 8 (2019).

[7] Martincová, J., Otyepka, M. & Lazar, P. Oxidation of metallic two-dimensional transition metal dichalcogenides: 1T-MoS2 and 1T-TaS2. *2D Mater.* **7**, 045005 (2020).

[8] Slot, T. K. *et al.* Surface oxidation of Ti3C2Tx enhances the catalytic activity of supported platinum nanoparticles in ammonia borane hydrolysis. *2D Mater.* **8**, 015001 (2020).

[9] Kistanov, A. A., Cai, Y., Zhou, K., Dmitriev, S. V. & Zhang, Y. W. Atomic-scale mechanisms of defect- and light-induced oxidation and degradation of InSe. *J. Mater. Chem. C* **6**, 518–525 (2018).

[10] Longo, R. C. *et al.* Intrinsic air stability mechanisms of two-dimensional transition metal dichalcogenide surfaces: Basal versus edge oxidation. *2D Mater.* **4**, 025050 (2017).

[11] Li, M., Li, T. & Jing, Y. Nb2S2C monolayers with transition metal atoms embedded at the S vacancy are promising single-atom catalysts for CO oxidation. *ACS Omega* **8**, 31051–31059 (2023). 37663518 10.1021/acsomega.3c02984 PMC10468833

[12] Dabral, A., Lu, A. K. A., Chiappe, D., Houssa, M. & Pourtois, G. A systematic study of various 2D materials in the light of defect formation and oxidation. *Phys. Chem. Chem. Phys.* **21**, 1089–1099 (2019). 30566131 10.1039/c8cp05665j

[13] Sun, T. *et al.* Defect chemistry in 2D materials for electrocatalysis. *Mater. Today Energy* **12**, 215–238 (2019).

[14] Joseph, T., Ghorbani-Asl, M., Batzill, M. & Krasheninnikov, V. Water dissociation and association on mirror twin boundaries in two-dimensional MoSe 2: Insights from density functional theory calculations. *Nanoscale Adv.* **3**, 6992–7001 (2021). 36132369 10.1039/d1na00429h PMC9419107

[15] Song, H. & Jiang, D. First principles insights into stability of defected MXenes in water. *Nanoscale* **15**, 16010–16015 (2023). 37672295 10.1039/d3nr02538a

[16] Gouveia, J. D. & Gomes, J. R. B. The determining role of tx species in the catalytic potential of MXenes: Water adsorption and dissociation on Mo2CTx. *Catal. Today* **424**, 113848 (2023).

[17] Hu, W., Li, Z. & Yang, J. Water on silicene: A hydrogen bond-autocatalyzed physisorption–chemisorption–dissociation transition. *Nano Res.* **10**, 2223–2233 (2017).

[18] Wang, Q. H., Kalantar-Zadeh, K., Kis, A., Coleman, J. N. & Strano, M. S. Electronics and optoelectronics of two-dimensional transition metal dichalcogenides. *Nat. Nanotechnol.* **7**, 699–712 (2012). 23132225 10.1038/nnano.2012.193

[19] Manzeli, S., Ovchinnikov, D., Pasquier, D., Yazyev, O. V. & Kis, A. 2D transition metal dichalcogenides. *Nat. Rev. Mater.* **2**, 1–15 (2017).

[20] Wang, L. *et al.* Intercalated architecture of MA2Z4 family layered Van Der Waals materials with emerging topological, magnetic and superconducting properties. *Nat. Commun.* **12**, 2361 (2021). 33883547 10.1038/s41467-021-22324-8 PMC8060390

[21] Tho, C. C. *et al.* MA2Z4 family heterostructures: Promises and prospects. *Appl. Phys. Rev.* **10**, 041307 (2023).

[22] Woźniak, T., Umm-e-hani, F., Junior, P. E., Ramzan, M. S. & Kuc, A. B. Electronic and excitonic properties of MSi2Z4 monolayers. *Small* **19**, 2206444 (2023). 10.1002/smll.202206444 36772899

[23] Ramzan, M. S., Woźniak, T., Kuc, A. & Cocchi, C. Composition-dependent absorption of radiation in semiconducting MSi_2_Z_4_ monolayers. *Phys. Status Solidi (b)*. 2300570. 10.1002/pssb.202300570 (2024).

[24] Hong, Y. L. *et al.* Chemical vapor deposition of layered two-dimensional MoSi2N4 materials. *Science* **369**, 670–674 (2020). 32764066 10.1126/science.abb7023

[25] Liu, H., Huang, B., Dai, Y. & Wei, W. Characteristic excitonic absorption of MoSi2N4 and WSi2N4 monolayers. *J. Phys. D: Appl. Phys.* **56**, 405103 (2023).

[26] Yao, H. *et al.* Novel two-dimensional layered MoSi2Z4 (Z = P, as): New promising optoelectronic materials. *Nanomaterials* **11**, 559 (2021). 33668165 10.3390/nano11030559 PMC7995989

[27] Xiao, C. *et al.* Adsorption behavior of environmental gas molecules on pristine and defective MoSi2N4: Possible application as highly sensitive and reusable gas sensors. *ACS Omega* **7**, 8706–8716 (2022). 35309471 10.1021/acsomega.1c06860 PMC8928539

[28] Liu, Y., Ji, Y. & Li, Y. Multilevel theoretical screening of novel two-dimensional MA2Z4 family for hydrogen evolution. *J. Phys. Chem. Lett.* **12**, 9149–9154 (2021). 34523936 10.1021/acs.jpclett.1c02487

[29] Lin, C. *et al.* Discovery of efficient visible-light driven oxygen evolution photocatalysts: Automated high-throughput computational screening of MA2Z4. *Adv. Funct. Mater.* **32**, 2207415 (2022).

[30] Qiu, D. Y., Jornada, D. & Louie, S. G. Optical spectrum of MoS 2: Many-body effects and diversity of exciton states. *Phys. Rev. Lett.* **111**, 216805 (2013). 24313514 10.1103/PhysRevLett.111.216805

[31] Eknapakul, T. *et al.* Electronic structure of a quasi-freestanding MoS2 monolayer. *Nano Lett.* **14**, 1312–1316 (2014). 24552197 10.1021/nl4042824

[32] Molina-Sánchez, A., Hummer, K. & Wirtz, L. Vibrational and optical properties of MoS2: From monolayer to bulk. *Surf. Sci. Rep.* **70**, 554–586 (2015).

[33] Joswig, J. O. *et al.* Mechanics, and energetics of two-dimensional MoS2 nanostructures from a theoretical perspective. *Acc. Chem. Res.* **48**, 48–55 (2015). 25489859 10.1021/ar500318p

[34] Singh, A. K., Mathew, K., Zhuang, H. L. & Hennig, R. G. Computational screening of 2D materials for photocatalysis. *J. Phys. Chem. Lett.* **6**, 1087–1098 (2015). 26262874 10.1021/jz502646d

[35] Ramzan, M. S., Bacic, V., Jing, Y. & Kuc, A. Electronic properties of a new family of layered materials from groups 14 and 15: First-principles simulations. *J. Phys. Chem. C* **123**, 25470–25476 (2019).

[36] Tawfik, S. A. *et al.* Efficient prediction of structural and electronic properties of Hybrid 2D materials using complementary DFT and machine learning approaches. *Adv. Theory Simul.* **2**, 1800128 (2019).

[37] Zhang, L., Wang, N. & Li, Y. Design, synthesis, and application of some two-dimensional materials. *Chem. Sci.* **14**, 5266–5290 (2023). 37234883 10.1039/d3sc00487b PMC10208047

[38] Bhattacharyya, S., Pandey, T. & Singh, A. K. Effect of strain on electronic and thermoelectric properties of few layers to bulk MoS2. *Nanotechnology* **25**, 465701 (2014). 25354843 10.1088/0957-4484/25/46/465701

[39] Mirabelli, G. *et al.* Air sensitivity of MoS2, MoSe2, MoTe2, HfS2, and HfSe2. *J. Appl. Phys.* **120**, 125102 (2016).

[40] Aggoune, W. *et al.* Dimensionality of excitons in stacked Van Der Waals materials: The example of hexagonal boron nitride. *Phys. Rev. B* **97**, 241114 (2018).

[41] Zhao, R. *et al.* External electric field and strains facilitated nitrogen dioxide gas sensing properties on 2D monolayer and bilayer SnS2 nanosheets. *Appl. Surf. Sci.* **491**, 128–137 (2019).

[42] Ramzan, M. S. & Cocchi, C. Strained MoTe2 monolayer as photon absorber in the telecom range (2023). 10.5281/zenodo.8221339 10.3390/nano13202740 PMC10608843 37887890

[43] Hohenberg, P. & Kohn, W. Inhomogeneous electron gas. *Phys. Rev.* **136**, B864–B871 (1964).

[44] Kresse, G. & Furthmüller, J. Efficient iterative schemes for *ab initio* total-energy calculations using a plane-wave basis set. *Phys. Rev. B* **54**, 11169–11186 (1996). 10.1103/physrevb.54.11169 9984901

[45] Blöchl, P. E. Projector augmented-wave method. *Phys. Rev. B* **50**, 17953–17979 (1994). 10.1103/physrevb.50.17953 9976227

[46] Perdew, J. P., Burke, K. & Ernzerhof, M. Generalized gradient approximation made simple. *Phys. Rev. Lett.* **77**, 3865–3868 (1996). 10062328 10.1103/PhysRevLett.77.3865

[47] Grimme, S., Antony, J., Ehrlich, S. & Krieg, H. A consistent and accurate ab initio parametrization of density functional dispersion correction (DFT-D) for the 94 elements H-Pu. *J. Chem. Phys.* **132**, 154104 (2010). 20423165 10.1063/1.3382344

[48] Liu, W. *et al.* Benzene adsorbed on metals: Concerted effect of covalency and Van Der Waals bonding. *Phys. Rev. B* **86**, 245405 (2012).

[49] Liu, W. *et al.* Structure and energetics of benzene adsorbed on transition-metal surfaces: Density-functional theory with Van Der Waals interactions including collective substrate response. *New. J. Phys.* **15**, 053046 (2013).

[50] Mallikarjun Sharada, S., Karlsson, R. K. B., Maimaiti, Y., Voss, J. & Bligaard, T. Adsorption on transition metal surfaces: Transferability and accuracy of DFT using the ADS41 dataset. *Phys. Rev. B* **100**, 035439 (2019).

[51] Birowska, M., Marchwiany, M. E., Draxl, C. & Majewski, J. A. Assessment of approaches for dispersive forces employing semihydrogenated graphene as a case study. *Comput. Mater. Sci.* **186**, 109940 (2021).

[52] Henkelman, G., Arnaldsson, A. & Jónsson, H. A fast and robust algorithm for Bader decomposition of charge density. *Comput. Mater. Sci.* **36**, 354–360 (2006).

[53] Momma, K. & Izumi, F. VESTA 3 for three-dimensional visualization of crystal, volumetric and morphology data. *J. Appl. Crystallogr.* **44**, 1272–1276 (2011).

[54] Sheoran, S., Monga, S., Phutela, A. & Bhattacharya, S. Coupled spin-valley, Rashba effect, and hidden spin polarization in WSi2N4 family. *J. Phys. Chem. Lett.* **14**, 1494–1503 (2023). 36745045 10.1021/acs.jpclett.2c03108

[55] Heyd, J., Scuseria, G. E. & Ernzerhof, M. Hybrid functionals based on a screened Coulomb potential. *J. Chem. Phys.* **118**, 8207–8215 (2003).

[56] Hedin, L. New method for calculating the one-particle Green’s function with application to the electron-gas problem. *Phys. Rev.* **139**, A796–A823 (1965).

[57] Qiu, P., Qin, Y., Bai, Y., Xia, Q. & Zheng, A. Gas selectivity regulation of monolayer SnS by introducing nonmetallic dopants: A combined theoretical and experimental investigation. *Appl. Surf. Sci.* **570**, 151155 (2021).

[58] Ramzan, M. S., Kuc, A. B. & Kim, H. S. Electronic fingerprint mechanism of NOx sensor based on single-material SnP3 logical junction. *npj Comput. Mater.* **8**, 1–10 (2022).

[59] Zhang, L. & Sit, P. H. L. Ab initio study of the role of oxygen and excess electrons in the degradation of CH_3_NH_3_PbI_3_. *J. Mater. Chem. A* **5**, 9042–9049 (2017).

[60] Peng, C., Chen, J., Wang, H. & Hu, P. First-principles insight into the degradation mechanism of CH_3_NH_3_PbI_3_ Perovskite: Light-Induced defect formation and water dissociation. *J. Phys. Chem. C* **122**, 27340–27349 (2018).

[61] Cai, M. Q., Busipalli, D. L., Xu, S. H., Nachimuthu, S. & Jiang, J. C. Exploring the air stability of all-inorganic halide perovskites in the presence of photogenerated electrons by DFT and AIMD studies. *Sustain. Energy Fuels* **6**, 3778–3787 (2022).

[62] Ramzan, M. S., Kunstmann, J. & Kuc, A. B. Tuning valleys and Wave functions of Van Der Waals Heterostructures by varying the number of layers: A first-principles study. *Small* 2008153. 10.1002/smll.202008153 (2021). 10.1002/smll.202008153 33955665

[63] Krumland, J. & Cocchi, C. Conditions for electronic hybridization between transition-metal dichalcogenide monolayers and physisorbed carbon-conjugated molecules. *Electron. Struct.* **3**, 044003 (2021).

[64] Peng, S., Cho, K., Qi, P. & Dai, H. Ab initio study of CNT NO2 gas sensor. *Chem. Phys. Lett.* **387**, 271–276 (2004).

[65] Chinh, N. D. *et al.* NO gas sensing kinetics at room temperature under UV light irradiation of In2O3 nanostructures. *Sci. Rep.* **6**, 35066 (2016). 27713526 10.1038/srep35066 PMC5054382

[66] Bafekry, A. *et al.* Adsorption of habitat and industry-relevant molecules on the MoSi2N4 monolayer. *Appl. Surf. Sci.* **564**, 150326 (2021).

[67] Szary, M. J. Toward high selectivity of sensor arrays: Enhanced adsorption interaction and selectivity of gas detection (N2, O2, NO, CO, CO2, NO2, SO2, AlH3, NH3, and PH3) on transition metal dichalcogenides (MoS2, MoSe2, and MoTe2). *Acta Mater.* **274**, 120016 (2024).

[68] Culp, J. T., Sui, L., Goodman, A. & Luebke, D. Carbon dioxide (CO2) absorption behavior of mixed matrix polymer composites containing a flexible coordination polymer. *J. Colloid Interface Sci.* **393**, 278–285 (2013). 23168045 10.1016/j.jcis.2012.10.050

[69] Illarionov, Y. Y. *et al.* The role of charge trapping in MoS2/SiO2 and MoS2/hBN field-effect transistors. *2D Mater.* **3**, 035004 (2016).

